# Long non-coding RNA SNHG8 enhances triple-negative breast cancer cell proliferation and migration by regulating the miR-335-5p/PYGO2 axis

**DOI:** 10.1186/s13062-021-00295-6

**Published:** 2021-08-06

**Authors:** Jintao Qian, Xinhan Lei, Yue Sun, Lu Zheng, Jia Li, Shuai Zhang, Lei Zhang, Wanwan Li, Jianing Shi, Wenjun Jia, Tong Tang

**Affiliations:** Department of General Surgery, the Second Affiliated Hospital of Anhui Medical University, Hefei, 230601 Anhui China

**Keywords:** Triple-negative breast cancer, SNHG8, miR-335-5p, PYGO2

## Abstract

**Background:**

Growing evidence has demonstrated that long non-coding RNAs (lncRNAs) can function as modulators in the development of triple-negative breast cancer (TNBC). However, the function of lncRNA small nucleolar RNA host gene 8 (SNHG8) in TNBC remains unclear. Therefore, our study aimed at investigating the role of SNHG8 in the proliferation and migration of TNBC cells.

**Methods:**

SNHG8 expression was evaluated using RT-qPCR assay. Cell proliferation and migration were assessed by EdU, colony formation and Transwell assays. The levels of proteins related to EMT process were examined by western blot assay. The interaction among SNHG8, miR-335-5p and pygopus family PHD finger 2 (PYGO2) was detected by RIP assay, RNA pull down assay and luciferase reporter assay.

**Results:**

SNHG8 expression was significantly up-regulated in TNBC cells. SNHG8 silencing obviously inhibited TNBC cell proliferation, migration and EMT process. Moreover, SNHG8 acted as a sponge to sequester miR-335-5p in TNBC cells. Besides, PYGO2 was proven as a target gene of miR-335-5p, and SNHG8 promoted TNBC cell proliferation, migration and EMT process through regulating miR-335-5p and PYGO2.

**Conclusions:**

Totally, our study indicated that SNHG8 promoted TNBC cell proliferation and migration by regulating the miR-335-5p/PYGO2 axis.

## Background

Breast cancer is one of the most common cancers in female worldwide [1]. The incidence of breast cancer occurs 25.1% of all cancers, and the mortality accounts for approximately 6.6% of global cancer death rate, making it the second cause of cancer-related death for women [2, 3]. Triple-negative breast cancer (TNBC) is a subtype of breast cancer and characterized by negative estrogen, progesterone receptors and proto-oncogene HER2 [4]. On account of high metastatic risk and lack of targeted therapy, the prognosis of patients with TNBC is poor [5]. As a consequence, a better understanding of the underlying mechanisms of TNBC progression may be conducive to the development of more novel and effective therapeutic strategies.

Long non-coding RNAs (lncRNAs) are generally classified as a category of RNAs longer than 200 nucleotides and cannot encode proteins [6, 7]. LncRNAs are involved in many biological processes ranging from housekeeping functions including transcription to more specific functions including genomic imprinting [8]. Of note, lncRNAs are also involved in a variety of activities such as the degradation and translation of messenger RNA or serving as RNA decoys or scaffolds [9]. Increasing evidences have demonstrated that the abnormal expression of lncRNAs can function as oncogenes or tumor suppressors to participate in the occurrence and development of cancers, including TNBC. For instance, overexpressed lncRNA GAS5 facilitates chemosensitivity and apoptosis of TNBC cells [10]. WT1-AS is with low expression in TNBC cells and exerts anticancer function in cell migration and invasion [11]. Moreover, some lncRNAs can function as potential prognostic biomarkers for TNBC [12]. LncRNA small nucleolar RNA host gene 8 (SNHG8) has been confirmed to exert carcinogenic function in many types of tumors. For instance, SNHG8 accelerates cell growth through sponging miR-663 in colorectal cancer cells [13]. ,SNHG8 promotes hepatocellular cancer tumorigenesis by sponging miR-149-5p [14]. SNHG8 acts as an oncogene of non-small-cell lung cancer via miR-542-3p to regulate CCND1/CDK6 [15]. However, the molecule mechanism and function of SNHG8 in TNBC remained unclear.

Therefore, the aim of our research was to investigate the function and detailed mechanism of SNHG8 in TNBC. We probed the expression of SNHG8 in TNBC and investigated the impacts of SNHG8 on TNBC cell proliferation, migration and epithelial-mesenchymal transition (EMT) process. Cytoplasmic lncRNAs are known as competing endogenous RNA (ceRNA) through sponging miRNAs in human cancers [16–18]. Here, we also explored whether SNHG8 played a similar role in TNBC.

Collectively, our study explored the function of SNHG8 and its downstream molecular mechanism in TNBC.

## Results

### SNHG8 facilitates the proliferation, migration and EMT process of TNBC cells

**Fig. 1:**
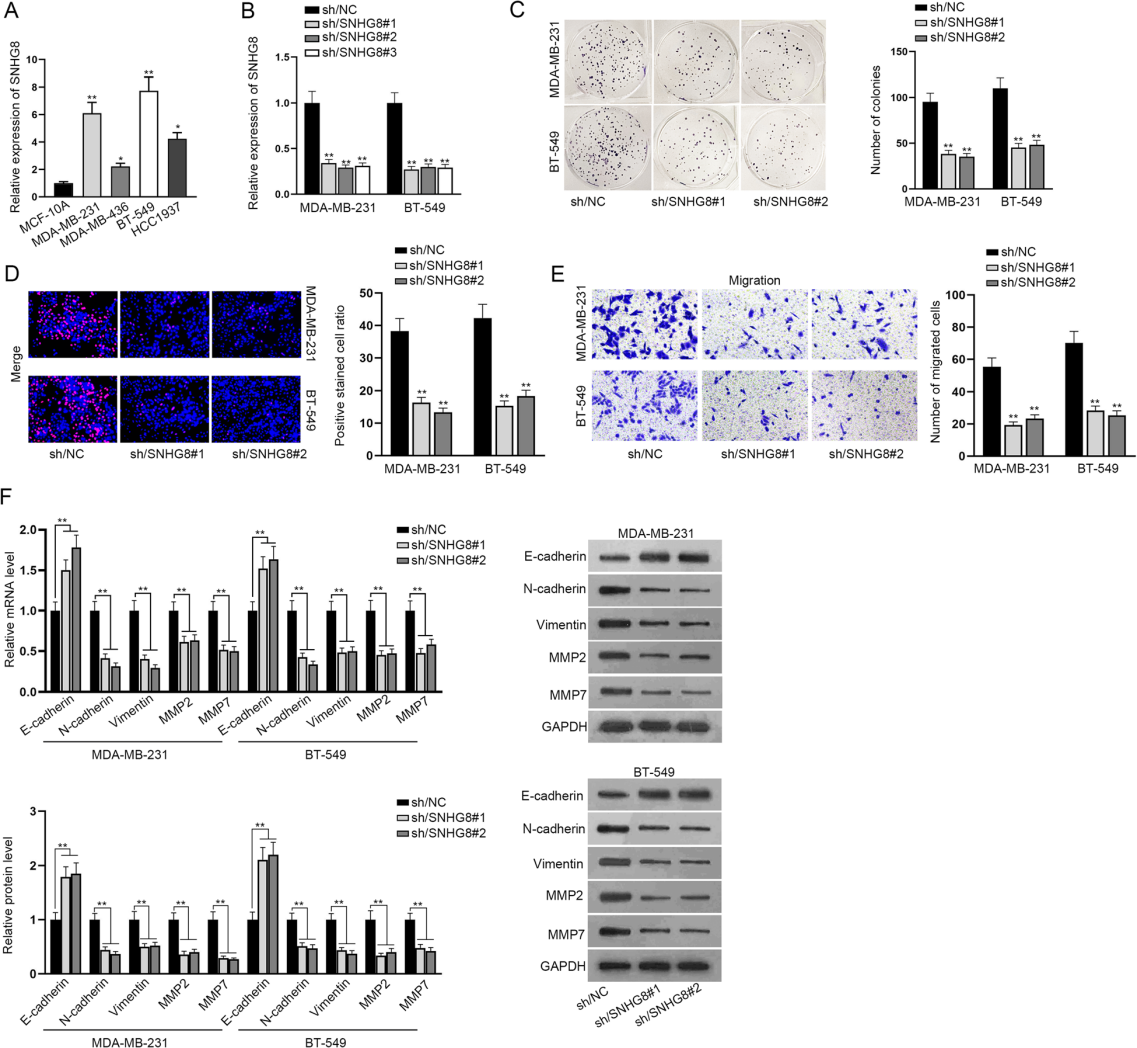
SNHG8 facilitates the proliferation, migration and EMT of TNBC cells. **A** The expression of SNHG8 was evaluated in four types of TNBC cells (MDA-MB-231, MDA-MB-436, BT-549 and HCC1937) and in normal breast epithelial cells (MCF-10A) by RT-qPCR assay. **B** RT-qPCR analysis indicated that SNHG8 expression was decreased in MDA-MB-231 and BT-549 cells respectively transfected with 3 shRNAs targeting SNHG8. **C**, **D** The influence of SNHG8 silencing on the proliferation of TNBC cells was examined using colony formation and EdU assays. **E** We detected the migratory capacity of TNBC cells affected by SNHG8 knockdown through Transwell assay. **F** RT-qPCR assay and western blot assay were adopted to analyze the E-cadherin, N-cadherin, Vimentin, MMP2 and MMP7 mRNA and protein levels with or without SNHG8 silencing. ^*^P < 0.05, ^**^P < 0.01

To basically understand the function of SNHG8 on the biological behaviors of TNBC cells, its expression in different cell lines was detected. We firstly evaluated SNHG8 expression in four common TNBC cell lines (MDA-MB-231, MDA-MB-436, BT-549 and HCC1937) compared with that in normal breast epithelial cells (MCF-10A). The result of RT-qPCR analysis indicated that SNHG8 expression was higher in each TNBC cell line than that in normal cells (Fig. 1A). To further explore whether SNHG8 influenced TNBC cell behaviors, the interference efficiency of SNHG8 was detected in MDA-MB-231 and BT-549 (which exhibited higher SNHG8 levels than other TNBC cells) through transfecting three shRNAs targeting SNHG8, respectively (Fig. 1B). According to the result of RT-qPCR assay, no significant difference was observed among the interference efficiency of sh/SNHG8#1, sh/SNHG8#2 and sh/SNHG8#3. Therefore, we selected sh/SNHG8#1 and sh/SNHG8#2 for the following assays. After that, the influence of SNHG8 silencing on cell growth and proliferation was inspected by colony formation assay and EdU assay. As shown by the results, the number of colonies was decreased by down-regulated SNHG8 (Fig. 1C). Similarly, SNHG8 silencing obviously inhibited the proliferative ability of TNBC cells (Fig. 1D). Besides, through Transwell assay, we observed that SNHG8 silencing impeded cell migratory capacity (Fig. 1E). Furthermore, as shown in Fig. 1F, SNHG8 knockdown increased E-cadherin mRNA and protein levels, while N-cadherin, Vimentin, MMP2 and MMP7 were down-regulated, suggesting that EMT process was repressed by SNHG8 depletion. Totally, SNHG8 played an oncogenic role in TNBC cells through facilitating the cell proliferation, migration and EMT process.

### SNHG8 serves as a sponge for miR-335-5p

**Fig. 2:**
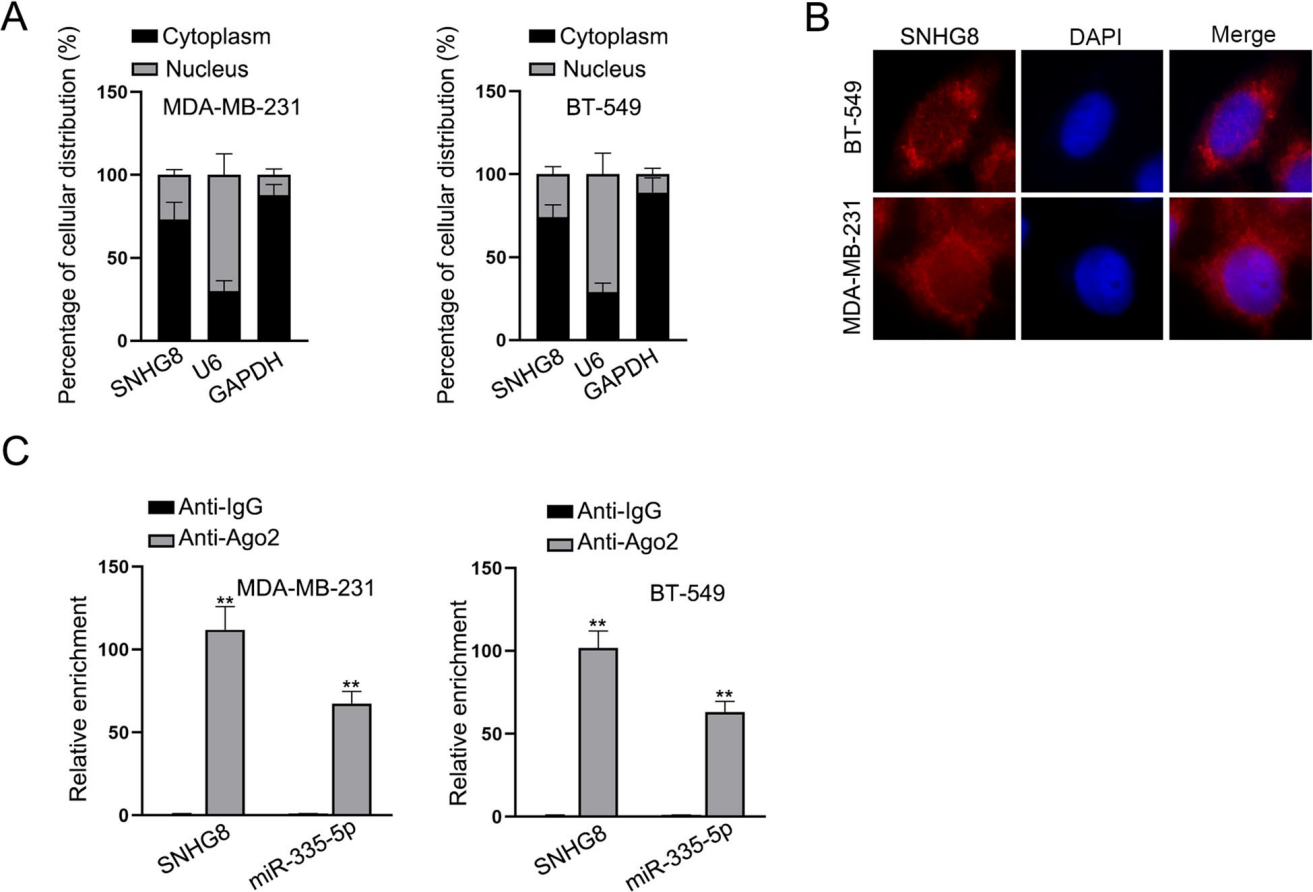
SNHG8 serves as a sponge for miR-335-5p. **A**, **B** Subcellular separation assay and FISH assay were conducted to confirm the distribution of SNHG8. **C** RIP experiment was conducted and it was verified that SNHG8 and miR-335-5p were enriched in the groups of Anti-Ago2. ^**^P < 0.01

To investigate the regulatory mechanism of SNHG8 in TNBC cells, subcellular separation assay and FISH assay were performed. As shown by the figures, it was exhibited that the major distribution of SNHG8 was in the cytoplasm (Fig. 2A and B). Moreover, by using ENCORI online bioinformatic tool (http://starbase.sysu.edu.cn), it was predicted that miR-335-5p could interact with SNHG8 under specific screening condition (Degradome-Data ≥1). Then, we conducted RIP assay in TNBC cells and found that both SNHG8 and miR-335-5p were highly enriched in Anti-Ago2 groups rather than in Anti-IgG groups, which indicated that they could coexist in TNBC cells (Fig. 2C). Taken together, SNHG8 was able to sequester miR-335-5p in TNBC cells.

### PYGO2 is a target gene of miR-335-5p

**Fig. 3:**
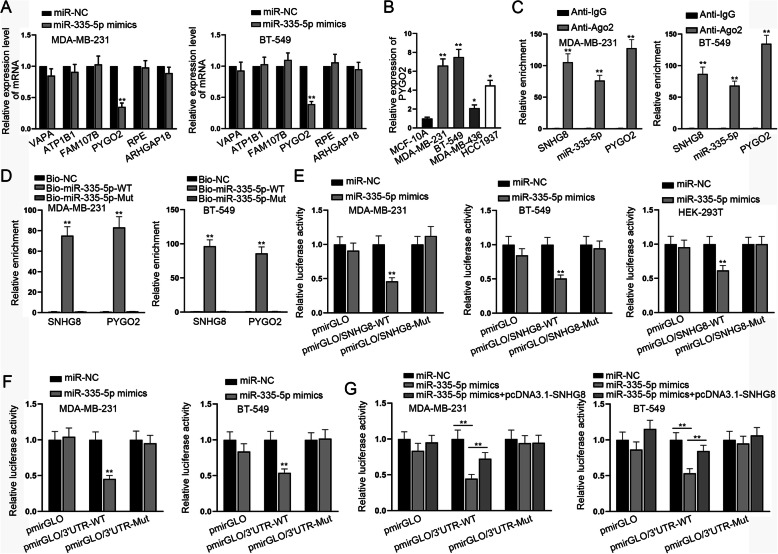
PYGO2 is the target gene of miR-335-5p. **A** The level changes of candidate genes were detected by RT-qPCR assay in TNBC cells when miR-335-5p was overexpressed. **B** PYGO2 expression in TNBC cells compared with that in normal cells was detected by RT-qPCR assay. **C** SNHG8, miR-335-5p and PYGO2 were significantly enriched in Anti-Ago2 groups, according to the result of RIP assay. **D** The binding capacity of SNHG8 or PYGO2 with miR-335-5p biotin probes was examined using RNA pull down assay. **E**, **F** As illustrated by luciferase reporter assay, the luciferase activities of pmirGLO/SNHG8-WT and pmirGLO/3’UTR-WT were reduced by miR-335-5p overexpression in MDA-MB-231, BT549 or HEK-293 T cells. **G** The luciferase activities of different reporters in TNBC cells respectively transfected with miR-NC, miR-335-5p mimics and miR-335-5p mimics + SNHG8 were separately detected. ^*^P < 0.05, ^**^P < 0.01

To determine the possible downstream genes of miR-335-5p, we utilized ENCORI and sifted out six candidate genes (VAPA, ATP1B1, FAM107B, PYGO2, RPE and ARHGAP18) which possessed possible binding ability to miR-335-5p under specific screening condition (Degradome-Data ≥2) according to TargetScan target-predicting program. Besides, according to the results of RT-qPCR, it was suggested that only PYGO2 was down-regulated in TNBC cells transfected with miR-335-5p mimics, thus we selected PYGO2 for further experiments (Fig. 3A). Then, RT-qPCR assay was carried out and it was demonstrated that PYGO2 expression was markedly elevated in several TNBC cells compared with that in MCF-10A cell line (Fig. 3B). Furthermore, according to the result of RIP assay, SNHG8, miR-335-5p and PYGO2 were significantly enriched in Anti-Ago2 groups, indicating that those three RNAs co-existed in RISCs (Fig. 3C). Moreover, by utilizing the RNA pull down assay, it was found that both SNHG8 and PYGO2 were obviously enriched in Bio-miR-335-5p-WT groups (Fig. 3D). Hence, it was certified that miR-335-5p could bind to both SNHG8 and PYGO2. Meanwhile, in MDA-MB-231, BT549 as well as HEK-293 T cells, luciferase reporter assay further demonstrated that miR-335-5p overexpression markedly reduced the luciferase activity of pmirGLO/SNHG8-WT or pmirGLO/3’UTR-WT, but there were no conspicuous changes in response to miR-335-5p mimics in mutant groups or normal control groups (Fig. 3E and F). Finally, we found that the luciferase activity of the PYGO2 3’UTR-WT group, which had been reduced after the overexpression of miR-335-5p, was partially rescued after SNHG8 up-regulation (Fig. 3G). Altogether, we found that in TNBC cells, PYGO2 was a target gene of miR-335-5p.

### SNHG8 promotes the proliferation, migration and EMT process of TNBC cells by sponging miR-335-5p

**Fig. 4:**
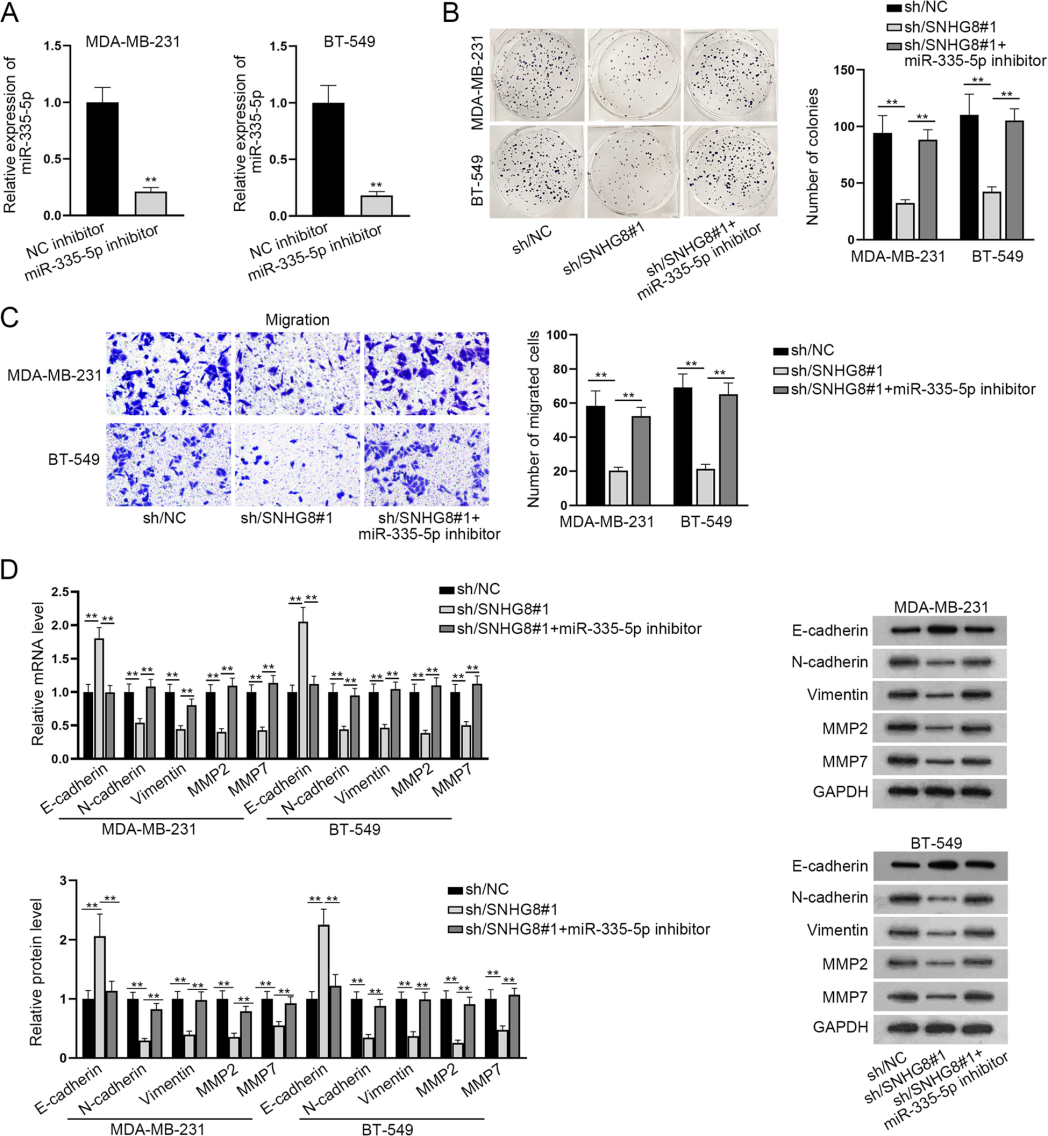
SNHG8 promotes the proliferation, migration and EMT of TNBC cells by sponging miR-335-5p. **A** We adopted RT-qPCR assay to detect miR-335-5p inhibitor transfection efficiency in TNBC cells. **B** Colony formation assay was conducted to detect the proliferation of TNBC cells transfected with sh/NC, sh/SNHG8#1 or sh/SNHG8#1 + miR-335-5p inhibitor. **C** Transwell assay was utilized to assess the number of migrated TNBC cells in different treatment groups. **D** RT-qPCR and western blot assays were used to examine E-cadherin, N-cadherin, Vimentin, MMP2 or MMP7 mRNA and protein levels in TNBC cells of each group. ^**^P < 0.01

Further, several rescue experiments were conducted to explore the relationship between miR-335-5p and SNHG8. First of all, through RT-qPCR assay, the interference efficiency of miR-335-5p was detected and a satisfactory result was observed (Fig. 4A). After that, through colony formation assay, we observed that TNBC cell proliferation reduced by SNHG8 silencing was enhanced after the co-transfection of miR-335-5p inhibitor (Fig. 4B). In addition, it was revealed in Transwell assay that SNHG8 knockdown reduced the number of migrated cells, while miR-335-5p depletion could partially reverse the suppressive effect (Fig. 4C). In addition, E-cadherin mRNA and protein levels were up-regulated by SNHG8 knockdown, and such result was partially reversed by miR-335-5p down-regulation. However, the levels of N-cadherin, Vimentin, MMP2 as well as MMP7 exhibited a contrary tendency (Fig. 4D). Totally, miR-335-5p was sponged by SNHG8, thus enhancing the proliferation, migration and EMT process of TNBC cells.

### The tumor-suppressive role of SNHG8 silencing on the proliferation, migration and EMT process of TNBC cells were reversed by PYGO2

**Fig. 5:**
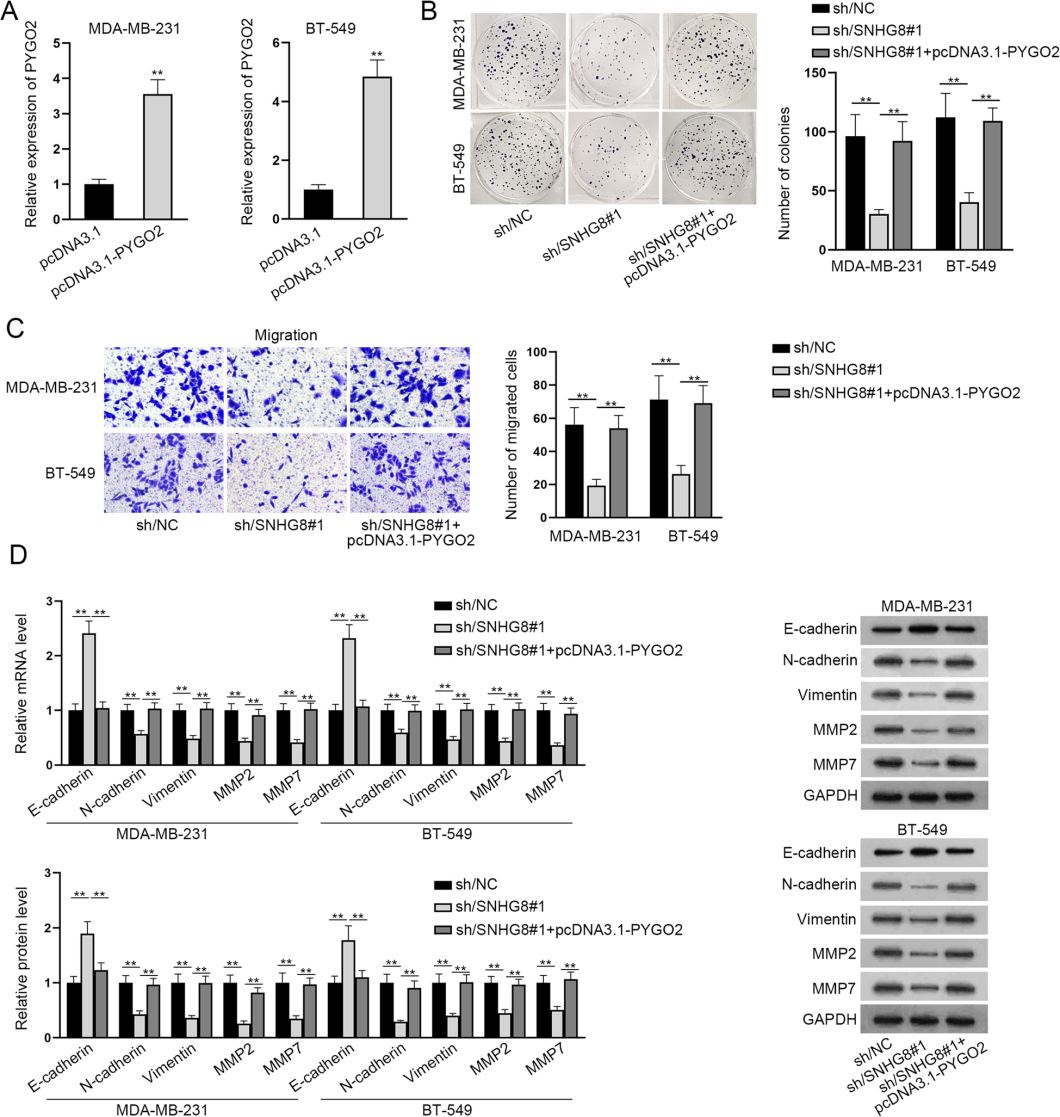
The tumor-suppressing role of SNHG8 silencing on the proliferation, migration and EMT process of TNBC cells were reversed by PYGO2. **A** RT-qPCR was used to detect PYGO2 overexpression efficiency in TNBC cells. **B** The proliferation of TNBC cells transfected with sh/NC, sh/SNHG8#1 or sh/SNHG8#1 + pcDNA3.1-PYGO2 was examined by colony formation assay. **C** Transwell assay was utilized to assess the number of migrated TNBC cells of sh/NC, sh/SNHG8#1 or sh/SNHG8#1 + pcDNA3.1-PYGO2 group. **D** RT-qPCR and western blot assays were conducted to examine E-cadherin, N-cadherin, Vimentin, MMP2 or MMP7 mRNA and protein levels in TNBC cells of sh/NC, sh/SNHG8#1 or sh/SNHG8#1 + pcDNA3.1-PYGO2 group. ^**^P < 0.01

Subsequently, we investigated the relationship between SNHG8 and PYGO2 in TNBC cells. After the transfection of pcDNA3.1-PYGO2 into TNBC cells, the overexpression efficiency of PYGO2 was detected and a satisfactory result was observed (Fig. 5A). In colony formation assay, SNHG8 silencing reduced the proliferative ability of TNBC cells, while the co-transfection of pcDNA3.1-PYGO2 could partially rescue the inhibitory effect (Fig. 5B). According to the result of Transwell assay, TNBC cell migration was weakened by SNHG8 down-regulation but the co-transfection of pcDNA3.1-PYGO2 could counteract the falling trend of migration (Fig. 5C). Through RT-qPCR and western blot assays, we found that E-cadherin mRNA and protein levels were increased by SNHG8 silencing but reduced after the co-transfection of pcDNA3.1-PYGO2. Besides, those of N-cadherin, Vimentin, MMP2 as well as MMP7 were suppressed in response to SNHG8 knockdown, but was partially reversed after the co-transfection of pcDNA3.1-PYGO2 (Fig. 5D). Together, those results indicated that SNHG8 contributed to TNBC cell proliferation, migration and EMT via regulating PYGO2.

## Discussion

A large number of studies have proven that the aberrant expression of lncRNAs is closely related to the progression of cancers. SNHG8 is up-regulated in pancreatic adenocarcinoma and the pancreatic adenocarcinoma patients with higher expression of SHNG8 are accompanied with shorter overall survival [19]. In addition, SNHG8 is highly expressed in esophageal squamous cell carcinoma and high SNHG8 expression is closely associated with TNM stage and worse overall survival among patients with esophageal squamous cell carcinoma [20]. Nevertheless, the role of SNHG8 in TNBC remains unclear. In our study, we found that SNHG8 expression was significantly elevated in TNBC cells, which suggested the oncogenic potential of SNHG8 in TNBC.

As reported previously, SNHG8 exerts oncogenic functions in the progression of many cancers. In gastric cancer, SNHG8 enhances cell proliferation and invasion [21]. In endometrial carcinoma, the knockdown of SNHG8 represses cell viability [22]. In colorectal cancer, SNHG8 accelerates the growth, migration and invasion of cells [13]. In line with those studies, our research also proved that SNHG8 deletion significantly impeded cell proliferation, migration and the EMT process of TNBC. All above results revealed the important role of SNHG8 in the malignant behaviors of TNBC, mirroring that SNHG8 might be a novel target for TNBC treatment.

Moreover, our study further found that SNHG8 could function as a ceRNA to exert functions at post-transcriptional level in TNBC cells. According to recent studies, SNHG8 contributes to the cell proliferation of osteosarcoma via binding to miR-542-3p [23]. Moreover, SNHG8 acts as a sponge of miR-149 to regulate the tumorigenesis and metastasis of hepatocellular carcinoma [14]. Consistently, our study further discovered that SNHG8 combined with miR-335-5p in TNBC cells. Moreover, rescue experiments confirmed that miR-335-5p inhibitor could partially reverse the inhibited effects of SNHG8 silence on TNBC proliferation, migration and the EMT process.

It is well documented that miRNAs function post-transcriptionally by usually base-pairing to the mRNAs 3′-untranslated regions to suppress protein synthesis [24]. It has been reported that miR-335-5p can inhibit thyroid cancer cells invasion and metastasis via targeting ICAM-1 [25]. In addition, miR-335-5p weakens colorectal cancer cell proliferation and migration via reducing LDHB [26]. In our study, we found that PYGO2 was the target gene of miR-335-5p in TNBC cells. Notably, PYGO2 has been proven to be up-regulated in breast cancer [27]. Here, we found that PYGO2 was up-regulated in TNBC cells and recovered SNHG8-mediated cellular function.

## Conclusion

In conclusion, our current study was the first in the field to reveal the role of SNHG8/miR-335-5p/PYGO2 pathway in TNBC cell proliferation, migration and EMT process. However, the lack of investigation on the upstream target of SNHG8 is a limitation of our current study. Therefore, we will further explore the upstream molecular mechanism of SNHG8 in our future study.

## Materials and methods

### Cell culture

Human normal breast epithelial cell line (MCF-10A), human TNBC cell lines (MDA-MB-231, MDA-MB-436, BT-549, HCC1937) as well as human embryonic kidney cell line (HEK-293 T) were all procured from the American Type Culture Collection (ATCC; Manassas, VA, USA) and preserved at 37 °C with 5% CO_2_. Leibovitz’s L-15 medium (Thomas Scientific, Swedesboro, NJ, USA) was applied to incubate MDA-MB-231 and MDA-MB-436 cells; ATCC-formulated RPMI-1640 medium (ATCC) was used for cultivating BT-549 and HCC1937 cells; Mammary Epithelial Cell Growth Medium (MEGM; Gibco, Grand Island, NY, USA) was adopted for cultivating MCF-10A cells; Dulbecco’s modified Eagle medium (DMEM; Gibco) was used for incubating HEK-293 T cells. 10% fetal bovine serum (FBS; Gibco) together with 1% penicillin-streptomycin (Sigma-Aldrich, St. Louis, MO, USA) was also applied to all media.

### Real-time quantitative polymerase chain reaction (RT-qPCR)

Total RNA in cultured cells was first extracted by using TRIzol Reagent (Invitrogen, Carlsbad, CA, USA). PrimeScript™ II Reverse Transcriptase (Takara, Kusatsu, Japan) was used for complementary DNA (cDNA) synthesis. CFX96 Touch Real-Time PCR Detection System (Bio-Rad, Hercules, CA, USA) together with SYBR Green PCR Kit (Takara) was applied to conduct PCR. All results were calculated by 2^−ΔΔCt^ method. The experiment was conducted for three times in an independent manner.

### Cell transfection

Confluent TNBC cells at 80–90% confluence were seeded into 6-well plates at 1 × 10^6^ cells/well for 48-h transfection with the specific short hairpin RNAs (shRNAs; GenePharma, Shanghai, China) against SNHG8 (sh/SNHG8#1 and #2), and nonspecific shRNAs (sh/NC) were used in negative control (NC) group. Besides, miR-335-5p mimics/inhibitor together with their relative NCs (miR-NC) were procured form RiboBio (Guangzhou, China). Overexpression of SNHG8 or PYGO2 was achieved by recombinant plasmids pcDNA3.1-SNHG8 or pcDNA3.1-PYGO2, and empty vectors were used as NC. Lipofectamine 3000 reagent (Invitrogen) was applied for cell transfection for 48 h.

### Colony formation

TNBC cells after transfection were seeded into 6-well cell culture plates (500 cells/well) and cultured for 14 days of colony formation. After that, colonies were immobilized by 4% paraformaldehyde, dyed by 0.5% crystal violet for 20 min and counted manually. The experiment was conducted for three times in an independent manner.

### 5-Ethynyl-2′ -deoxyuridine (EdU) assay

After the 48-h transfection, TNBC cells after transfection were laid in 96-well plates (1 × 10^4^ cells/well) and labeled using BeyoClick™ EdU Cell Proliferation Kit (Beyotime, Shanghai, China). Cell nuclei visualization was conducted with the aid of 4′,6-diamidino-2-phenylindole (DAPI) staining solution. Eventually, a fluorescence microscope (Olympus, Tokyo, Japan) was used for observing positively labeled cells. The experiment was conducted for three times in an independent manner.

### Transwell migration assay

TNBC cells after transfection were planted in the upper chamber of Transwell chambers (24-well; Corning Incorporated, Corning, NY, USA). Complete culture medium, meanwhile, was filled in the lower chamber which was cultivated with PBS. Twenty-four hours later, cells migrating to the lower chamber were removed with caution by a cotton swab and then fixed in methanol solution for 15 min. Crystal violet was adopted to stain the membranes for 10 min, and the invaded or migrated cells were observed and counted under a microscope (Olympus) at the magnification of 10 × 10. The experiment was conducted for three times in an independent manner.

### Subcellular separation assay

Cytoplasmic & Nuclear RNA Purification Kit was commercially acquired from Norgen Biotek Corp (Thorold, ON, Canada) for conducting subcellular separation assay in TNBC cells according to the instruction from the provider. Cells were treated in cell fractionation buffer to isolate cell cytoplasm. SNHG8 levels in cytoplasmic and nuclear fractions were separately detected by RT-qPCR, using GAPDH and U6 as controls. The experiment was conducted for three times in an independent manner.

### Fluorescence in situ hybridization (FISH) assay

RNA FISH probe specific to SNHG8 was designed and synthesized at RiboBio and utilized as per the instruction. Air-dried cells were incubated with probes in hybridization buffer, and DAPI was adopted to observe nuclei. Images were obtained using a fluorescence microscope (Olympus). The experiment was conducted for three times in an independent manner.

### RNA immunoprecipitation (RIP) assay

Magna RIP™ RNA-Binding Protein Immunoprecipitation Kit (Millipore, Billerica, MA, USA) was used as per the instruction. Cell lysates and Ago2 antibody (1:50; MA5–14861; Invitrogen) conjugated on magnetic beads were co-cultured in RIP buffer overnight at 4 °C through rotation, while IgG antibody (1:500; 31,786; Invitrogen) was utilized as NC. Finally, the RNA precipitates were purified and extracted using the Imprint® RNA Immunoprecipitation Kit (RIP-12RXN, Sigma-Aldrich, USA) and RT-qPCR was then applied for relative RNA enrichment examination. The experiment was conducted for three times in an independent manner.

### RNA pull down assay

Pierce Magnetic RNA-Protein Pull-Down Kit (Thermo Scientific, Waltham, MA, USA) was applied for RNA pull down assay according to the standard method from the provider. Protein extracts were collected and incubated with biotinylated miR-335-5p probe, followed by the addition of streptavidin magnetic beads. Then RNAs were eluted down the beads and analyzed using RT-qPCR. The experiment was conducted for three times in an independent manner.

### Luciferase reporter gene assay

SNHG8 sequence or the 3′-untranslated region (3′-UTR) of PYGO2 with the predicted binding sequence for miR-335-5p was cloned into pmirGLO dual-luciferase reporter gene vectors (Promega, Madison, WI, USA), and pmirGLO/SNHG8-WT and pmirGLO/3’UTR-WT plasmids were thus obtained. Meanwhile, pmirGLO/SNHG8-Mut and pmirGLO/3’UTR-Mut plasmids were constructed using SNHG8 or the PYGO2 3’UTR sequence with mutant binding sequence for miR-335-5p. Luciferase reporter plasmids and other specific plasmids were co-transfected into cells for 48 h, and Dual-Luciferase® Reporter Assay System (Promega) was then applied to examine relative luciferase activity (firefly/Renilla). Assay was performed three times.

### Statistical analyses

All quantitative assays were bio-repeated thrice, and experimental results were displayed as mean ± standard deviation (SD). A *p*-value less than 0.05 was considered as statistically significant. GraphPad PRISM 6 (GraphPad, La Jolla, CA, USA) was used for analyzing data. Group difference comparison was conducted with Student’s *t*-test or one-way/two-way analysis of variance (ANOVA).

## Data Availability

Not applicable.

## Abbreviations

lncRNA: Long non-coding RNA
ceRNA: Competing endogenous RNA
mRNA: Messenger RNA
miRNA: MicroRNA
shRNA: Short hairpin RNA
UTR: Untranslated region
TNBC: Triple-negative breast cancer
SNHG8: Small nucleolar RNA host gene 8
PYGO2: Pygopus family PHD finger 2
EMT: Epithelial-mesenchymal transition
ATCC: American Type Culture Collection
FBS: Fetal Bovine Serum
FISH: Fluorescence in situ hybridization
RT-qPCR: Real-time quantitative polymerase chain reaction
EdU: 5-ethynyl-2′-deoxyuridine
DAPI: 4′,6-diamidino-2-phenylindole
SDS-PAGE: Sodium dodecyl sulfate-polyacrylamide gel electrophoresis
PVDF: Polyvinylidene difluoride
RIP: RNA immunoprecipitation
RISC: RNA-induced silencing complex
GAPDH: Glyceraldehyde-3-phosphate dehydrogenase
WT: Wild-type
Mut: Mutant
NC: Negative control
SD: Standard deviation
ANOVA: Analysis of variance
